# Habitat structure is linked to the evolution of plumage colour in female, but not male, fairy-wrens

**DOI:** 10.1186/s12862-016-0861-3

**Published:** 2017-01-26

**Authors:** Iliana Medina, Kaspar Delhey, Anne Peters, Kristal E. Cain, Michelle L. Hall, Raoul A. Mulder, Naomi E. Langmore

**Affiliations:** 1 Division of Ecology and Evolution, Research School of Biology, Australian National University, Canberra, 0200 Australia; 2 School of Biological Sciences, Monash University, Melbourne, Australia; 3 Max Planck Institute for Ornithology, Radolfzell, Germany; 4 School of Biological Sciences, University of Auckland, Auckland, New Zealand; 5 School of BioSciences, University of Melbourne, Melbourne, VIC 3010 Australia

**Keywords:** Colour, Ornamentation, Female, Fairy-wren, Conspicuousness, Sexual dimorphism, Crypsis

## Abstract

**Background:**

Both natural and sexual selection may drive the evolution of plumage colouration in birds. This can lead to great variation in plumage not only across species, but also between sexes within species. Australasian fairy-wrens are famous for their brightly coloured males, which exhibit colours ranging from bright blue to red and black. Female plumage in fairy wrens (and in general) has been rarely studied, but it can also be highly variable, including both bright and cryptic plumages. We use a comparative framework to explore the basis for this variation, and test the possibility that female fairy-wrens experience selection for cryptic plumage when they occupy more exposed habitats that offer little concealment from predators. We use spectral measurements of plumage for species and subspecies of Australasian fairy-wrens.

**Results:**

We show that female colouration (contrast against background) is strongly correlated with vegetation cover: females in open habitats show less contrast to background colours than females in closed habitats, while male colouration is not associated with habitat type.

**Conclusions:**

Female plumage appears to be under stronger natural selection than male plumage in fairy-wrens, providing an example of how selection may act differently on males and females of the same species.

**Electronic supplementary material:**

The online version of this article (doi:10.1186/s12862-016-0861-3) contains supplementary material, which is available to authorized users.

## Background

Birds famously show great variation in their plumage colouration, and this variation is thought to be the product of a range of selective pressures. Conspicuous colours are considered to be useful for intra-specific communication, such as competition for mates, resources, and courtship [1–5], while selection for crypsis and camouflage may favour the evolution of less conspicuous colours [6, 7]. There is evidence that plumage colouration can vary greatly in response to pressures associated with habitat type [6, 8]. For instance, species of warblers (*Phylloscopus* spp.) with brighter markings live in darker habitats [9]. Bright and contrasting colours may be favoured in dark habitats, to maximize conspicuousness to conspecifics [9], while dull and pale colours may be favoured in open habitats to maximize crypsis, since predation rates may be elevated when vegetation cover is reduced [10–14].

The interplay between sexual and natural selection may result in the evolution of differences in colour between species as well as differences between the sexes in the same species [15]. In several species, cryptic plumage appears to have evolved in more exposed habitats in one sex only. For instance, in Eclectus parrots (*Eclectus rotatus*), females are secure inside their nest hollow for up to 11 months/year and have evolved bright red plumage that advertises nest hollow ownership, whereas males spend most of their time foraging in the rainforest canopy and have evolved cryptic, green plumage [16]. Similarly, in the barn owl (*Tyto alba*) there is selection for cryptic plumage in open habitats, but this is true only for females and not for males [10]. By contrast, bright plumage has been sexually selected in male grackles (Icteridae), but only in open habitats [17]. Also, Drury & Burroughs [18] found a link between how exposed nests were (e.g. a proxy for predation risk) and sexual dichromatism across new world blackbirds, where higher predation risk was associated with higher differences in colour between males and females. All these studies suggest that natural selection plays an important role in generating plumage colour differences between males and females. However, contrary to what had been previously thought, phylogenetic analyses now show that sexual dichromatism across species can often result from changes in plumage in females, not males [15, 19–21]. Selection on females may be important in explaining colour differences between males and females.

Several studies suggest that the selective pressures behind the evolution of plumage in birds are different for males and females [15, 22]. Males and females often have different pathways to fitness, hence the relative costs and benefits of bright plumage in each sex are also likely to change according to habitat [23, 24]. Australasian fairy-wrens and emu-wrens (AVES: Maluridae) are widely recognized for their bright and conspicuous colouration. Males of many species and females of a few species exhibit patches of striking structurally-based plumage (e.g. blue) or bright red to orange carotenoid-based colouration [25]. Fairy-wrens occur in a wide range of habitats, from rainforest in New Guinea to grasslands and deserts in Australia, and there is high variation in plumage not only across species and subspecies, but also between males and females [22]. In fairy-wrens, it has been proposed that sexual selection pressures may have driven the evolution of conspicuous colours in male fairy-wrens and the closely related emu-wrens [22, 26]. However, the evolution of colouration in female fairy-wrens has received less attention. The available studies, based on qualitative colour assessments, show a positive link between latitude and dichromatism; relatively duller females and conspicuous males are usually, but not always, found at higher latitudes [22]. Johnson et al. [22] suggested that environmental differences associated with changes in latitude could play an important role in the evolution of female colouration. For instance, variables that change with the latitudinal gradient, such as habitat structure or seasonality could be important in determining how conspicuous individuals are.

In this study we use a comparative framework to test for the first time whether habitat characteristics, specifically vegetation cover, can explain the observed variation in colour and contrast among fairy-wren species with special emphasis on female colouration. Fairy-wrens are an ideal group to study the evolution of plumage, because, although there is high variation in colouration, all fairy and emu-wren species have similar life-history traits (e.g. group living, non-migratory, dome nesting). We measured plumage reflectance in more than 600 museum specimens and used published vegetation indices to calculate a principal component that describes vegetation cover in each of the distributions of these species (or subspecies). Using this information, we test whether differences in vegetation cover can explain 1. The level of contrast of plumage against common natural backgrounds and 2. The percentage of structural (usually bright) colouration. We predicted that, if there is selection for increased crypsis, less conspicuous phenotypes will be found in more open habitats, where species are more exposed to visual predators. We test this hypothesis for both males and females.

## Results

The principal component used to describe vegetation cover (99% of variation explained) was significantly correlated to latitude (*r* ^2^ = 0.44, *p* < 0.001), to the average annual rainfall in each location (*r* ^2^ = 0.44, *p* = 0.045) but not average temperature (*r* ^2^ = 0.02, *p* = 0.91). We used this principal component for all posterior analyses.

**Fig. 1:**
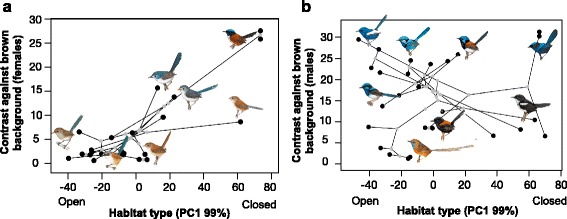
Association between habitat type (vegetation cover) and the contrast of the female (**a**) and male (**b**) head plumage against a brown background. Phylogenetic relationships are represented by branches and grey points represent ancestral states connecting black points (tips). Results remain qualitatively similar when the contrast is against a green background. Drawings of species made by Hilary Burn and used with permission from del Hoyo et al. (2014)

Colouration was measured in males and females in seven body patches. Contrast between the colour and two common backgrounds was calculated for both males and females using visual modelling. In females, vegetation cover was positively and significantly related to the amount of contrast for the head, throat and cheeks, after controlling for the effect of latitude and regardless of the type of background or phylogenetic tree used (e.g. green or brown background, Table 1, and achromatic contrast, 0.039 to 0.265, *N* = 23 species and subspecies, Fig. 1a). The heads, throats and cheeks of females from species in open habitats contrasted less with backgrounds than those of females from species in closed habitats. Contrast of the back was also positively correlated with the amount of vegetation cover, but only when latitude (non-significant) was removed from the model (β =0.007 to 0.088). Since vegetation cover is significantly correlated with latitude (*r* ^2^ = 0.7) we believe that the lack of association between back contrast and vegetation cover when latitude is present could be a statistical artefact, due to the collinearity of the predictor variables. This means that there is a link between latitude (or habitat) and back contrast but we cannot be sure which one is driving this association. Contrast of the breast, belly and tail was not correlated with vegetation coverage or latitude (Table 1). Overall, effects were stronger when using the brown than the green background. The phylogenetic signal of contrast against background varied greatly between phylogenies (λ mean: 0.15, 95% HDP: 0.0001 to 0.31).

**Table 1:**
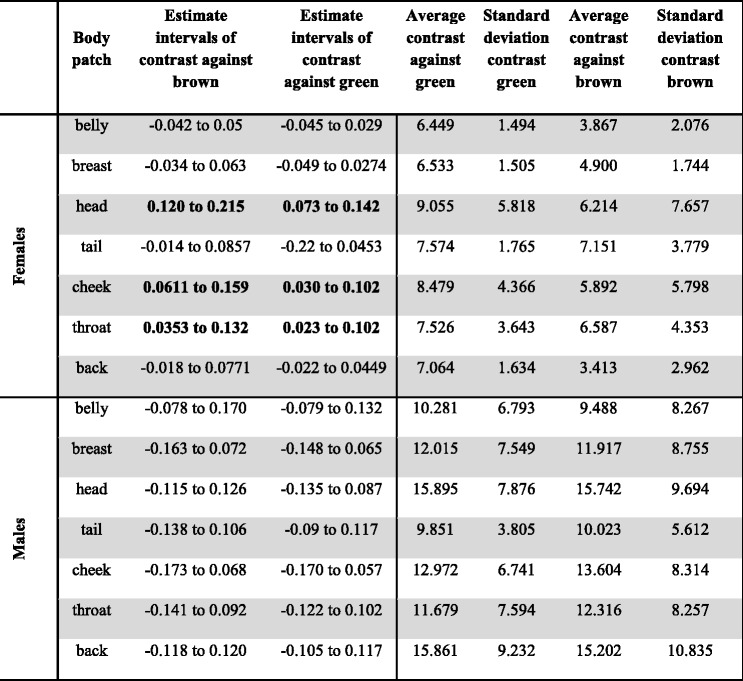
Results of association between habitat type (e.g. vegetation cover PC) and different measures of colour contrast for different body patches in females (top) and males (bottom), against green and brown backgrounds. Intervals represent variation in β (estimate) across phylogenetic trees. A significant association between colouration and habitat type is considered when the intervals presented do not overlap with zero (shown in bold). Average level of contrast per patch in JNDs and standard deviation is also shown for 23 taxa in females and 26 taxa in males

For males, there was no association between vegetation cover and the level of contrast of the species in any of the patches (Fig. 1b, all intervals overlapped with zero, *r* ^2^ = 0.129 for contrast against green and *r* ^2^ = 0.11 for contrast against brown). The phylogenetic signal in this association was also very low (λ mean: 0.08, 95% HDP between 0.0001 and 0.17). On average, all patches in males were more contrasting than the patches in females (Table 1), but there was also greater variation in contrast in males. The least contrasting patches in females were the belly and the breast, and in males the tail and the belly.

**Fig. 2:**
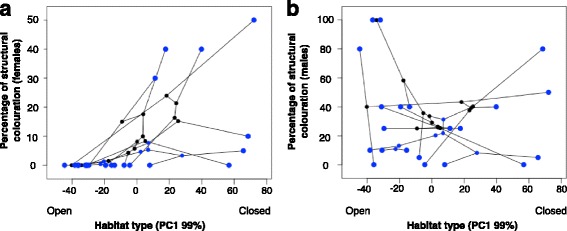
Association between habitat type (vegetation cover) and the percentage of structural colour in the body of females (**a**) and males (**b**) for different species. Phylogenetic relationships are represented by branches and black points represent ancestral states connecting blue points (tips)

Females had a lower percentage of structural colours than males (8.33% vs. 29.75% respectively). The percentage of the body covered by structurally coloured plumage was significantly and positively associated with vegetation cover in females (β =0.23 to 0.25, Fig. 2), but not males (β = −0.21 to 0.07). Females in open environments had a lower percentage of their body covered by structural colours.

## Discussion

The idea that sexual selection has driven the evolution of elaborate colouration and ornamentation in male birds is a well-established paradigm in animal behaviour [2, 15, 27], but the evolution of female bird colouration has received much less attention [27]. It has been shown [22] that latitude could explain variation in colouration in female fairy-wrens. However, relationships with latitude are usually a result of associations with other variables that vary latitudinally, such as habitat or seasonality. Our aim here was to determine more precisely whether variation in colouration could be explained by differences in habitat structure across species. We show that, for female fairy-wrens and emu-wrens, species and subspecies living in open habitats are less likely to exhibit plumage colours that contrast less with the background colour of the environment than species living in closed habitats. The percentage of the body covered by structural colouration was also lower in species living in open habitats. We found no such pattern in males, suggesting sex-specific selective pressures. Thus natural, rather than sexual selection, may be a stronger force driving colour evolution in female fairy-wrens.

We found that in female fairy-wrens and emu-wrens, habitat was a strong predictor of head, throat, cheek (and back) plumage contrast, and that species in closed habitats had more contrasting body patches. This suggests not only that the balance of natural and sexual selection pressures is different for males and females, but also that natural and sexual selection may interact in different ways on different body patches. In females, patches that showed a higher association with vegetation type were mostly dorsal, and they were also the most contrasting ones (e.g. head, cheeks and back). On the other hand, ventral patches such as the belly and breast presented overall lower contrast and lower variation (lower SD in Table 1) across species and the lack of an effect in these patches maybe due to lack of variation in contrast (e.g. most species and subspecies are dull). Where the primary predators are aerial, dorsal patches (such as head and back) are likely to be most strongly influenced by natural selection, whereas facial/frontal patches are likely to be most relevant for intra-specific communication [28] and thus more influenced by inter- and intra-sexual selection [29–31]. While contrasting facial patches in female fairy-wrens could function in intra-specific communication, natural selection appears to have selected against conspicuousness in these patches in species that live in open habitats. Our results highlight the value of analysing different components of plumage, because it enables detection of anatomically localised patterns, which may be missed if body plumage is assumed to evolve as a whole [32].

Our finding that female conspicuous colouration was more common in closed habitats corresponds with that of Marchetti [9] in *Phylloscopus* warblers, where species breeding in darker and closed habitats had brighter colours than those breeding in open habitats. However, Marchetti [9] argued that dull birds evolve in open habitats because birds are easily visible to each other even without the presence of bright colours. Accordingly, social selection favours changes in plumage colouration in order to maintain adequate and similar levels of visibility (e.g. contrast) [9]. Contrary to this, we found that the contrast of female fairy-wrens to background colours changed among habitats. Species that live in closed habitats have higher contrast against the background (either green or brown) while species in open habitats tend to be less contrasting. Our findings do not support a scenario where selection for conspicuousness leads to plumage variation. Instead, our results could suggest a scenario where selection favours cryptic phenotypes in open habitats. Predation risk might be higher in such habitats due to greater exposure to visual predators [11, 12]. It has been shown in other systems that colour (e.g. conspicuousness) can significantly increase predation rates [33, 34]. Parental care at the nest might make it more advantageous for females to have cryptic plumage and might constrain plumage brightness if females spend more time at the nest than males [35]. Moreover, because male plumage varies seasonally while female plumage is constant year-round, conspicuousness might impose higher costs on females [36]. Strong selection for crypsis in females could have led to the reduction of conspicuous colouration, and it may be that the sexually selected benefits of expressing colourful breeding plumage in males are much greater than the naturally selected costs. It would be interesting to test whether non-breeding plumage in male-fairy wrens is also subject to the same predation pressures as that of female fairy-wrens. Hofmann et al.[20] showed that in new world orioles (*Icterus* sp.) elaborate plumages have been consistently lost in females through evolutionary history, with convergent selection for more cryptic plumages. Our results show a similar pattern, although the low phylogenetic signal in our analyses prevents us from making inferences about the ancestral colouration in female fairy-wrens. Loss of bright plumage has also been observed in ducks (although in males), and this has resulted in loss of sexual dichromatism in many duck species [37]. Another interesting possibility is that predators with different visual systems might be prevalent to different degrees in types of habitats, and this could drive plumage differences between habitats [13]. In open habitats, raptors (with good colour vision) could be an important predation pressure, while in closed habitats mammals may represent a larger threat. Mammals have lower sensitivity to ultraviolet light [38], and thus animals with UV patches could be inconspicuous to mammalian predators.

Given that our measure of ‘vegetation cover’ was a composite of several environmental layers, it is also possible that this variable encompasses other habitat characteristics, besides the openness of the environment. For instance, this variable was also correlated with higher precipitation and measures of plant productivity. Producing structural plumage is presumed to be costly [1, 39, 40] (although see Prum [41]). Areas with reduced plant productivity may have more limited food resources (fairy- and emu-wrens are insectivorous [25]). If females benefit less than males from expressing structural colouration, then female trait expression may be reduced in resource-poor environments. The sexual selection benefits of expressing structural colouration could still make it worth expressing this trait in males, but not in females in poor environments. Under this scenario, environmental productivity, and not predation rates, would be responsible for the evolution of plumage in female fairy-wrens. In blackbirds it has been suggested that increased productivity in marshes may have led to brighter colour patches, although in such case these were all carotenoid-based colourations, not structural [42].

## Conclusions

We provide evidence suggesting that the evolution of conspicuous colouration in female, but not male, fairy-wrens is tightly linked to the habitat type. Our findings are consistent with previous suggestions regarding the selective pressures influencing the evolution of plumage colour in fairy-wrens, where selection to maximize conspicuousness to conspecifics may be driving the evolution of male colouration [43], while natural selection may constrain the evolution of female colouration. This constitutes an important addition to the body of literature suggesting that female and male ornamentation can be under different selective pressures, and that both natural and sexual selection are important drivers of sexual dichromatism. Our results also reveal differential evolution of colour patches, and show that different parts of the body may be under different types of selective pressures. We encourage future experiments to test whether females in open habitats suffer higher predation pressures, which might lead to increased selection for cryptic plumage in open environments. Moreover, it would be interesting to explore whether more contrasting females within a population actually suffer higher levels of predation.

## Methods

### Phylogenetic and geographic information

We downloaded 1300 different possible phylogenies from birdtree.org [44] for 15 species of fairy-wren and emu-wren (*Malurus spp*., *Stipiturus spp*. and *Clytomyias insignis*, see full list in Additional file 1: Figure S1). These phylogenies were sampled from a posterior distribution of possible phylogenies and some of the trees have the same topology of phylogenetic hypotheses previously suggested for the Maluridae [45]. By taking all of these potential phylogenies into account we ensure that our results are consistent irrespective of which are the real phylogenetic relationships. Since the trees available in birdtree.org do not contain information on subspecies, we added nine subspecies (details of subspecies used in Additional file 1) as the closest taxa to the nominal species, and included an arbitrary branch length of 0.025, considering that the time since divergence for subspecies of *Malurus* sp. has been very recent (<220.000 years ago [46]). Analyses for a branch length set to 0.05 gave qualitatively identical results.

We collected information on geographic distribution from the Atlas of Living Australia and birdlife.org. We downloaded shape files with the distribution of each species and subspecies. To calculate differences in habitat we downloaded three raster files with information on Evapotranspiration from the Wisconsin Center for Sustainability and the Global Environment (nelson.wisc.edu [47]), Net Primary Productivity [48], and a potential vegetation index [49], which describes the vegetation that would exist in a given location had human forms of land use not existed. We extracted the average values of each raster file for each area of distribution of each species or subspecies using the free software QGIS [50]. After obtaining an average value per raster and per species we generated a principal component using the three different rasters and covariance matrices. The three environmental layers used were highly correlated, and the principal component obtained from this analysis represents 99% of the variation (Additional file 1: Figure S2) and it is directly related to the amount of area covered by trees and vegetation. Thus, higher values represent areas with higher vegetation density, such as tropical evergreen forests, and low values represent open areas with less vegetation, such as grasslands, shrublands and savannahs. From these shape files we also calculated the mean latitude of the distribution of each taxon. The environmental layers used do not capture changes in vegetation across seasons. Nevertheless, the inclusion of latitude captures the possible effect of seasonality, while the vegetation layer represents biomes and more obvious and consistent differences in habitat across locations. We also aimed to collect body size variables for each taxon, however, these were unavailable for many species, and for those available, body size ranges largely overlap, suggesting that there are very little differences in size within this clade, with most fairy-wren species being described as being between 7 and 12 g, except for the larger *M. cyanocephalus* (12 to 17 g) [51].

### Contrast analysis

To calculate the level of plumage contrast for males and females from each species we measured plumage reflectance of museum specimens in the bird-visible wavelength range (300–700 nm) using a reflectance spectrometer (Avaspec 2048, Avantes, Eerbeek, The Netherlands) connected to a xenon pulsed light source (Avalight-Xe, Avantes, Eerbeek, The Netherlands) via a bifurcated fibre optic cable. The measuring end of the fibre optic cable was fitted with a black plastic cylinder to standardise measuring distance and exclude ambient light. Reflectance spectra were collected by gently pressing the measuring probe against the plumage at an angle of 90°, and reflectance was computed relative to a WS-2 white standard. We measured seven plumage patches (head, back, breast, belly, cheek, throat and tail) belonging to female and male fairy-wren and emu-wren specimens housed at the Australian National Wildlife Collection in Canberra and the Melbourne Museum, Australia, although in some specimens not all plumage patches could be measured due to damage. Damaged, dirty or dishevelled plumage was not measured. Depending on size and condition we computed between 1–2 reflectance spectra per plumage patch and specimen, and in total we obtained 7480 spectra belonging to 23 taxa for females and 26 for males, and an average of 16 individuals per sex per taxon. Some subspecies for which spectral data was available were not used, since there was no precise information on the geographic distribution (e.g. environmental layers) or phylogenetic relationships.

Reflectance spectra were down-sampled to 5 nm steps and imported into the R statistical environment. We used visual models [52] as implemented in Cassey et al. [53] using R scripts described in Delhey et al. [54]. Visual models compute a set of xyz coordinates that define the position of each reflectance spectrum in the three-dimensional colour space of birds. Differences in colouration between two reflectance spectra are represented by distances and their unit is the just noticeable difference (JND), whereby a difference below 1 JND is deemed not to be discriminable [55]. Visual models require knowledge on the visual sensitivity functions of the different photoreceptors used for colour vision, the signal-to-noise ratio of these photoreceptors and the spectrum of the ambient light.

Most birds have four types of single cones that are used in colour vision, sensitive to long (L), medium (M), short (S) and very short (VS) wavelengths of light. Interspecific variation in visual sensitivities is largely restricted to the VS and S cones and most birds fall into one of two groups, violet-sensitive species (V-type), which are less UV sensitive than ultraviolet sensitive species (U-type) (Cuthill, 2006). Fairy-wrens are unusual among birds in that closely related species differ in their visual sensitivity: some have V-type and some U-type vision [56]. Here we use average V-type visual sensitivity functions (from Appendix A in Endler & Mielke, [57] because our aim is to assess how conspicuous fairy-wrens are to their main visual predators, birds of prey, which have V-type vision. The noise-to-signal ratio of each cone type (ω_L_ = 0.05; ω_M_ = 0.046; ω_S_ = 0.06; ω_VS_ = 0.08) was calculated using formula 10 in Vorobyev et al. [55], average cone proportions from [58] (0.38:0.69:1.14:1;VS:S:M:L) based on a Weber fraction of 0.05 for the L cone [55]. Finally, we used the irradiance spectrum of standard daylight (d65 [55]) as illuminant.

Chromatic coordinates (xyz) for each reflectance spectrum obtained from visual models were averaged within a patch, so that we had one set of coordinates per patch per individual and then averaged between individuals of the same sex to obtain one set of mean chromatic coordinates per patch per sex per taxon (species/subspecies). These chromatic coordinates were used to compute the Euclidean distance between each plumage patch in every species and the chromatic coordinates of two common types of natural backgrounds (green leaves and brown bark or soil). Background spectra were obtained from Delhey et al.[43] and processed using the same visual models as described above. These Euclidean distances indicate to what extent the different plumage patches resemble natural backgrounds in colour, with higher values indicating more dissimilar, contrasting and, most likely, conspicuous colours. For each species we averaged these values of contrast against both types of backgrounds to obtain a single value of overall conspicuousness for each taxon studied.

Given that for fairy-wrens the brightest, most conspicuous and common type of colouration is given by structural colours (found in this study), we also quantified the percentage of the body covered by structural colouration. To calculate this, we first obtained information on which patches of each species comprise structural colouration, based on the spectral data obtained from museum specimens, following the procedure described in [59]. Based on this information, we divided the body into seven different regions (head, back, breast, belly, cheek, throat and tail) and calculated the proportion of regions that had UV reflective colouration. The estimation of area covered by structural colouration was calculated using images from plates from a field guide [25].

### Statistical analyses

To test the association between plumage contrast and habitat type we used a phylogenetic mixed model approach (MCMCglmm) in the R package MCMCglmm [60]. We controlled for phylogenetic non-independence by including 1300 different phylogenies in the model. To correct for phylogenetic uncertainty we followed Ross et al. [61] and sampled a tree from the posterior distribution of trees at iteration t, running the MCMC mixed model for 1000 iterations and saving the last sample. This process was repeated for 1300 iterations and we disposed the first 300 runs as burn-in. The predictor variables of the model were a principal component describing habitat type, the interaction between habitat type and colour patch (e.g. part of the body measured) and latitude. The response variables were contrast against green or brown background and achromatic contrast. The phylogenetic information and species identity were used as random factors. We collected estimates for each iteration and then generated a High Density Probability (HDP) interval of 95% for each parameter. If intervals overlapped with zero, we considered that there was no significant effect of the predictor on the amount of plumage contrast. We used the function *phylomorphospace* in the phytools R package [62] to plot our results, which provides information on the phylogenetic relationships between species/subspecies. A similar model was used to test the association between percentage of structural colouration and habitat type.

## Additional files

Additional file 1: List of species used in the study, example of phylogeny used in the analyses and results of principal component analysis. (DOCX 318 kb)
Additional file 2: Dataset contrast: Raw values of three environmental layers for each subspecies, the principal component used in the analysis (pc1_veg) and values of contrast against green background (cont.g) and brown background (cont.b). Page one contains values for females and page two contains values for males. (XLS 106 kb)

## References

[1] Siefferman L, Hill GE (2005). UV-blue structural coloration and competition for nestboxes in male eastern bluebirds. Anim Behav.

[2] Hill GE (1993). Male mate choice and the evolution of female plumage coloration in the house finch. Evolution.

[3] Hill GE, McGraw KJ (2006). Bird coloration: mechanisms and measurements.

[4] Hegyi GB, Rosivall B, Szollosi E, Hargitai R, Eens M, Torok J (2007). A role for female ornamentation in the facultatively polygynous mating system of collared flycatchers. Behav. Ecol..

[5] Murphy TG, Rosenthal MF, Montgometie RD, Tarvin KA (2009). Female American goldfinches use carotenoid-based bill coloration to signal status. Behav Ecol.

[6] Gomez D, Théry M (2004). Influence of ambient light on the evolution of colour signals: comparative analysis of a Neotropical rainforest bird community. Ecol Lett.

[7] Endler J (1990). On the measurement and classification of colour in studies of animal colour patterns. Biol J Linn Soc.

[8] McNaught MK, Owens IPF (2002). Interspecific variation in plumage colour among birds: species recognition or light environment. J Evol Biol.

[9] Marchetti K (1993). Dark habitats and bright birds illustrate the role of the environment in species divergence. Nature.

[10] Dreiss AN, Antoniazza S, Burri R, Fumagalli L, Sonnay C, Frey C, Goudet J, Roulin A (2011). Local adaptation and matching habitat choice in female barn owls with respect to melanic coloration. J Evol Biol.

[11] Hughes JJ, Ward D (1993). Predation risk and distance to cover affect foraging behaviour in Namib desert gerbils. Anim Behav.

[12] Moreno S, Villafuerte R, Delibes M (1996). Cover is safe during the day but dangerous at night: the use of vegetation by European wild rabbits. Can J Zool.

[13] Boinski S, Kauffman L, Westoll A, Stickler CM, Cropp S, Ehmke E (2003). Are vigilance, risk from avian predators and group size consequences of habitat structure? A comparison of three species of squirrel monkey (*Saimiri oerstedii, S. boliiensis* and *S. sciureus*). Behaviour.

[14] Huhta E, Mappes T, Jokimaki J (1996). Predation on artificial ground nests in relation to forest fragmentation, agricultural land and habitat structure. Ecography.

[15] Dale J, Dey CJ, Delhey K, Kempenaers B, Valcu M (2015). The effects of life history and sexual selection on male and female plumage colouration. Nature.

[16] Heinsohn R, Legge S, Endler J (2005). Extreme reversed sexual dichromatism in a bird without sex role reversal. Science.

[17] Eaton M (2006). A phylogenetic perspective on the evolution of chromatic ultraviolet plumage coloration in grackles and allies (Icteridae). The Auk.

[18] Drury JP, Burroughs N (2015). Nest shape explains variation in sexual dichromatism in New World blackbirds. J. Avian Biol..

[19] Dunn PO, Armenta JK, Whittingham LA (2015). Natural and sexual selection act on different axes of variation in avian plumage color. Sci Adv.

[20] Hofmann CM, Cronin TW, Omland KE (2008). Evolution of sexual dichromatism. 1. Convergent losses of elaborate female coloration in New World Orioles (Icterus spp.). The Auk.

[21] Price JJ, Eaton MD (2014). Reconstructing the evolution of sexual dichromatism: current color diversity does not reflect past rates of male and female change. Evolution.

[22] Johnson A, Price JJ, Pruett-Jones S (2013). Different modes of evolution in males and females generate dichromatism in fairy-wrens (Maluridae). Ecol Evol.

[23] Stuart-Fox D, Ord TJ (2004). Sexual selection, natural selection and the evolution of dimorphic coloration and ornamentation in agamid lizards. Proc. R. Soc.

[24] Cain KE, Rosvall KA (2014). Next steps for understanding the selective relevance of female-female competition. Front Ecol Evol.

[25] Rowley I, Russell E. Fairy-wrens and grasswrens: Maluridae, vol. 4. Oxford: Oxford University Press; 1997.

[26] Friedman NR, Remes V (2015). Rapid evolution of elaborate male coloration is driven by visual system in Australian Fairy-wrens (Maluridae). J Evol Biol.

[27] Amundsen T (2000). Why are female birds ornamented?. Trends Ecol. Evol..

[28] Green JD, Osmond LH, Double CM, Cockburn A (2000). Display rate by male fairy-wrens (Malurus cyaneus) during the fertile period of females has little influence on extra-pair mate choice. Behav Ecol Sociobiol.

[29] Järvi T, Bakken M (1984). The function of the variation in the breast stripe of the great tit *(Parus major)*. Anim Behav.

[30] Linville SU, Breitwisch R, Schilling AJ (1998). Plumage brightness as an indicator of parental care in northern cardinals. Anim Behav.

[31] Rutowski RL, Nahm AC, Macedonia JM (2010). Iridiscent hindwing patches in the Pipevine Swallowtail: differences in dorsal and ventral surfaces relate to signal function and context. Funct. Ecol..

[32] Smith KR, Cadena V, Endler JA, Porter WP, Kearney MR, Stuart-Fox D (2016). Colour change on different body regions provides thermal and signalling advantages in bearded dragon lizards. Proc. R. Soc. Lond. B Biol. Sci..

[33] Stuart-Fox D, Moussalli A, Marshall AJ, Owens IPF (2003). Conspicuous males suffer higher predation risk: visual modelling and experimental evidence from lizards. Anim Behav.

[34] Godin JJ, McDonough HE (2002). Predator preference for brightly colored males in the guppy: a viability cost for a sexually selected trait. Behavioral Ecology.

[35] Martin TE, Badyaev AV (1996). Sexual dichromatism in birds: Importance of nest predation and nest location for females versus males. Evolution.

[36] Peters A, Kingma SA, Delhey K (2013). Seasonal male plumage as a multicomponent sexual signal: insights and opportunities. Emu.

[37] Omland KE (1997). Examining two standard assumptions of ancestral reconstructions: repeated loss of dichromatism in Dabbling ducks (Anatini). Evolution.

[38] Honkavaara J, Koivula M, Korpimäki E, Siitari H, Viitala J (2002). Ultraviolet vision and foraging in terrestrial vertebrates. OIKOS.

[39] Eliason CM, Shawkey MD (2011). Decreased hydrophobicity of iridiscent feathers: a potential cost of shiny plumage. J. Exp. Biol..

[40] McGraw KJ, Mackillop EA, Dale J, Hauber ME (2002). Different colors reveal different information: how nutritional stress affects the expression of melanin- and structurally based ornamental plumage. J. Exp. Biol..

[41] Prum RO, Hill GE, McGraw KJ (2006). Anatomy, physics, and evolution of structural colors. Bird coloration: mechanisms and measurements.

[42] Johnson KP, Lanyon SM (2000). Evolutionary changes in color patches of blackbirds are associated with marsh nesting. Behav. Ecol..

[43] Delhey K, Hall ML, Kingma SA, Peters A (2013). Increased conspicuousness can explain the match between visual sensitivities and blue plumage colours in fairy-wrens. Proc. R. Soc.

[44] Jetz W, Thomas G, Hartmann A, Mooers O (2012). The global diversity of birds in space and time. Nature.

[45] Lee JY, Joseph L, Edwards SV (2012). A species tree for the Australo-Papuan fairy-wrens and allies (Aves: Maluridae). Syst Biol.

[46] Driskell A, Pruett-Jones S, Tarvin KA, Hagevik S (2003). Evolutionary relationships among blue- and black-plumaged populations of the white-winged fairy-wren (*Malurus leucopterus*). Aust. J. Zool.

[47] Willmott CJ, Matsuura K (2001). Terrestrial water budget data archive. Monthly time series (1950–1999).

[48] Kucharik CJ, Foley JA, Delire VA, Fisher MT, Coe J, Lenters J, Young-Molling N, Ramankutty N, Norman JM, Gower ST (2000). Testing the performance of a dynamic global ecosystem model: water balance, carbon balance and vegetation structure. Global Biogeochem Cycles.

[49] Ramankutty N, Foley JA (1999). Estimating historical changes in land cover: North American croplands from 1850 to 1992. Glob Ecol Biogeogr.

[50] QGIS (2015). Quantum GIS geographic information System. Open Source Geospatial Foundation Project.

[51] del Hoyo J, Elliott A, Sargata J, Christie DA, de Juana E (2014). Handbook of the birds of the world alive.

[52] Vorobyev M, Osorio D (1998). Receptor noise as a determinant of colour thresholds. Proc. R. Soc.

[53] Cassey P, Ewen JG, Blackburn TM, Hauber ME, Vorobyev M, Marshall NJ (2008). Eggshell colour does not predict measures of maternal investment in eggs of *Turdus* thrushes. Naturwissenschaften.

[54] Delhey K, Delhey V, Kempenaers B, Peters A (2015). A practical framework to analyze variaton in animal colors using visual models. Behav Ecol.

[55] Vorobyev M, Osorio D, Bennett ATD, Marshall NJ, Cuthill I (1998). Tetrachromacy, oil droplets and bird plumage colours. J Comp Physiol A.

[56] Ödeen A, Pruett-Jones S, Driskell A, Armenta JK, Håstad O (2012). Multiple shifts between violet and ultraviolet vision in a family of passerine birds with associated changes in plumage coloration. Proc. R. Soc.

[57] Endler JA, Mielke PW (2005). Comparing entire colour patterns as birds see them. Biol J Linn Soc.

[58] Hart NS (2001). The visual evology of avian photoreceptors. Prog Retin Eye Res.

[59] Delhey K (2015). The colour of an avifauna: a quantitative analysis of the colour of Australian birds. Sci Rep.

[60] Hadfield JD (2010). MCMC methods for multi-response generalized linear mixed models: The MCMCglmm R package. J. Stat. Softw..

[61] Ross L, Gardner A, Hardy N, West SA (2013). Ecology, not the genetics of sex determination, determines who helps in Eusocial populations. Curr Biol.

[62] Revell LJ (2012). phytools: an R package for phylogenetic comparative biology (and other things). Methods Ecol. Evol..

