# Supplementary material for: Habitat structure is linked to the evolution of plumage colour in female, but not male, fairy-wrens
Source: BMC Evol Biol. 2017 Jan 26;17:35. doi: 10.1186/s12862-016-0861-3 (PMC5270345; doi:10.1186/s12862-016-0861-3)
Supplement: Additional file 1: — List of species used in the study, example of phylogeny used in the analyses and results of principal component analysis. (DOCX 318 kb) [file 12862_2016_861_MOESM1_ESM.docx]

**Additional file 1**

**Table S1.** Species and subspecies used for the comparative analyses, values for vegetation cover layers and proportions of structural (str) colouration calculated.

| **Species** | **Subspecies** | **Net primary productivity** | **Vegetation cover** | **Evapotranspiration** | **Male str** | **Fem str** |
| --- | --- | --- | --- | --- | --- | --- |
| *Clytomyias_insignis* | oorti | 1.24 | 6.131 | 68.96 |  |  |
| *Malurus_alboscapulatus* | moretoni | 1.06 | 2.709 | 117.73 | 0 | 0 |
| *Malurus_amabilis* | amabilis | 0.52 | 8.19 | 78.94 | 25 | 40 |
| *Malurus_coronatus* | coronatus | 0.493 | 9.227 | 61.79 | 5 | 0 |
| *Malurus_coronatus* | macgillivrayi | 0.36 | 10.04 | 54.335 |  |  |
| *Malurus_cyaneus* | cyaneus | 1.102 | 6.949 | 49.09 | 40 | 0 |
| *Malurus_cyaneus_cyano* | cyanochlamys | 0.4978 | 8.0246 | 47.504 |  |  |
| *Malurus_cyanocephalus* | bonapartii | 1.079 | 2.174 | 129.48 |  |  |
| *Malurus_cyanocephalus* | cyanocephalus | 1.079 | 2.174 | 129.48 | 80 | 10 |
| *Malurus_elegans* | elegans | 0.72 | 8.86 | 41.89 | 40 | 0 |
| *Malurus_lamberti* | dulcis | 0.4 | 9.154 | 72.52 | 25 | 30 |
| *Malurus_lamberti* | lamberti | 0.2 | 10.59 | 31.69 | 25 | 0 |
| *Malurus_leucopterus* | leuconotus | 0.167 | 10.7 | 16.7 | 80 | 0 |
| *Malurus_melanocephalus* | cruentatus | 0.308 | 9.91473 | 58.4206 |  |  |
| *Malurus_melanocephalus* | melanocephalus | 0.5483 | 8.199 | 62.9083 | 0 | 0 |
| *Malurus_pulcherrimus* | pulcherrimus | 0.38 | 10.45 | 30.19 | 40 | 0 |
| *Malurus_splendens* | melanotus | 0.21 | 10.24 | 29.43 |  |  |
| *Malurus_splendens* | callainus | 0.17 | 10.86 | 24.76 | 100 | 0 |
| *Stipiturus_malachurus* | littleri | 1.1186 | 7.16129 | 48.7802 |  |  |
| *Stipiturus_malachurus* | malachurus | 0.7817 | 6.5725 | 47.53975 | 10 | 0 |
| *Stipiturus_malachurus* | westernensis | 0.561 | 9.88 | 36.144 |  |  |
| *Stipiturus_mallee* | mallee | 0.31 | 10.53 | 30.55 | 10 | 0 |
| *Stipiturus_ruficeps* | ruficeps | 0.17 | 11.42 | 23.05 | 10 | 0 |
| *Clytomyias_insignis* | insignis | 1.24 | 6.131 | 68.96 | 0 | 0 |
| *Malurus_campbelli* | campbelli | 1.085 | 6.131 | 100.975 | 40 | 40 |
| *Malurus_grayi* | grayi | 1.085 | 1.27 | 132.99 | 50 | 50 |
| *Sipodotus_wallacii* | wallacii | 1.081 | 2.18 | 126.58 | 5 | 5 |
| *Malurus_pulcherrimus* | pulcherrimus | 0.38 | 10.45 | 30.19 | 40 | 0 |
| *Malurus_leucopterus* | leucopterus | 0.2 | 12 | 25.53 | 0 | 0 |

**Figure S1.** Example of phylogeny used for contrast analyses with subspecies included as the closest taxa to nominal species.

**Figure S2.** Association between environmental layers and principal components. The PC1 explains 99.9% of the variation in these three layers and it is positively correlated with the amount of evapotranspiration in the location and with net primary productivity. It is negatively related to the ‘vegetation type’ layer.
